# Soemmerring’s Rings Developed around IOLs, in Human Donor Eyes, Can Present Internal Transparent Areas

**DOI:** 10.3390/ijms232113294

**Published:** 2022-10-31

**Authors:** Justin Christopher D’Antin, Francesc Tresserra, Rafael I. Barraquer, Ralph Michael

**Affiliations:** 1 Institut Universitari Barraquer, Universitat Autònoma de Barcelona, 08021 Barcelona, Spain; 2 Centro de Oftalmología Barraquer, 08021 Barcelona, Spain; 3 Department of Pathology, Institut Universitari Dexeus, 08028 Barcelona, Spain; 4 Department of Medicine, Faculty of Medicine and Health Sciences, Universitat Internacional de Catalunya, 08017 Barcelona, Spain; 5 Institute for Medical Informatics, Statistics, and Epidemiology (IMISE), Leipzig University, 04109 Leipzig, Germany

**Keywords:** Soemmerring’s ring, IOL, lens, transparency, histology

## Abstract

Soemmerring’s rings consist of a ring of lens epithelial derived cells that grow along the periphery of an aphakic lens capsule, or around an intraocular lens. These rings when visualized frontally, appear opaque, however, in some cases the cells that compose these rings are organized in the same fashion as those in normal transparent adult lenses. Thus, our purpose was to test whether any part of the adult Soemmerring’s ring could be transparent and how this related to morphological factors. To study this, 16 Soemmerring’s rings were extracted from donor eye globes. After imaging, they were thickly sectioned sagittally in order to analyze the degrees of transparency of different areas. All samples were also histologically analyzed using alpha smooth muscle actin, Vimentin, wheat germ agglutinin and DAPI. Our results showed that many samples had some transparent areas, mostly towards the center of their cross-section. Of the factors that we analyzed, only lens fiber organization at the bow region and an increased area of mature lens fiber cells had a significant relation to the degree of transparency at the center. Thus, we can conclude that as Soemmerring’s rings mature, they can develop organized and transparent areas of lens cells.

## 1. Introduction

Soemmerring’s rings [1] are a result of the proliferation and aberrant fiber differentiation of residual lens epithelial cells that remain in the lens capsule after cataract surgery, with or without intraocular lens (IOL) implantation [2]. They are a type of posterior capsule opacification, consisting of a ring of cells and tissue that develop and grow within the lens capsule along the equator.

In the case of Soemmerring’s rings developed around IOLs, since they develop outside of the visual axis, there is no need to remove them unless they cross the mechanical barrier of the IOL and disrupt vision. Even in these cases, the growths on or behind the IOL are only cleared from the visual axis by creating a posterior laser capsulotomy. This process will clear the visual axis, but still leave the areas around the IOL intact, allowing the Soemmerring’s ring to continue developing.

When these Soemmerring’s rings are extracted from ex-vivo donor globes, they mostly appear opaque and milky. Despite this, when sagittally sectioned for histological analysis in many cases very organized lens fiber layers can be observed [3].

In our previous article [3], we observed that the morphology of some samples was comparable to normal transparent lenses. However, despite these well-organized lens fiber cells, all our Soemmerring’s rings when viewed frontally with dark field illumination, appeared opaque.

The relationship between lens fiber cell organization and lens transparency has been well documented and studied [4,5,6]. In 2008 Michael et al. showed the relation between cortical cataract opacifications and lens fiber disorder or damage. They showed how wrinkles and tears in the normally straight and layered lens fibers, correlated directly to the opacifications and distortions visible in cortical cataracts [5].

In 2009, Donaldson et al. studied how the disruption of the lens fiber order leads to light scatter and eventually cataracts. They showed that the volume of the fiber cells that make up the bulk of the lens needs to be tightly regulated if lens transparency is to be preserved [4].

Interestingly, partially transparent Soemmerring’s rings have previously been observed, induced and reported in rabbits and human infants [7,8,9,10,11,12]. In these cases, the lens was extracted through a smaller than normal (6–7 mm) anterior rhexis size which ranged from 4.5–5; 2–2.5 and even 1–1.5 mm in diameter. It was shown that, a reduced anterior rhexis size promotes proper lens fiber differentiation and lens regeneration. This was probably due to various factors, such as the increased preservation of the majority of the lens epithelial cells under the anterior capsule. The fact that after lens extraction, the anterior capsule tends to adhere to the posterior capsule and a smaller rhexis size closes and heals more completely. This closure leads to a complete occlusion of the capsule opening, separating the lens material from the anterior chamber, creating a sealed microenvironment which largely prevents inflammatory cytokines from entering the capsule, decreasing epithelial to mesenchymal transformation.

However, the possibility of transparency in normal adult Soemmerring’s rings, grown around IOLs, has not been properly studied.

Thus, our objective is to test whether any part of these adult Soemmerring’s rings is transparent, despite appearing opaque when viewed frontally, and how this could relate to morphological factors.

## 2. Results

### 2.1. Transparency

**Figure 1:**
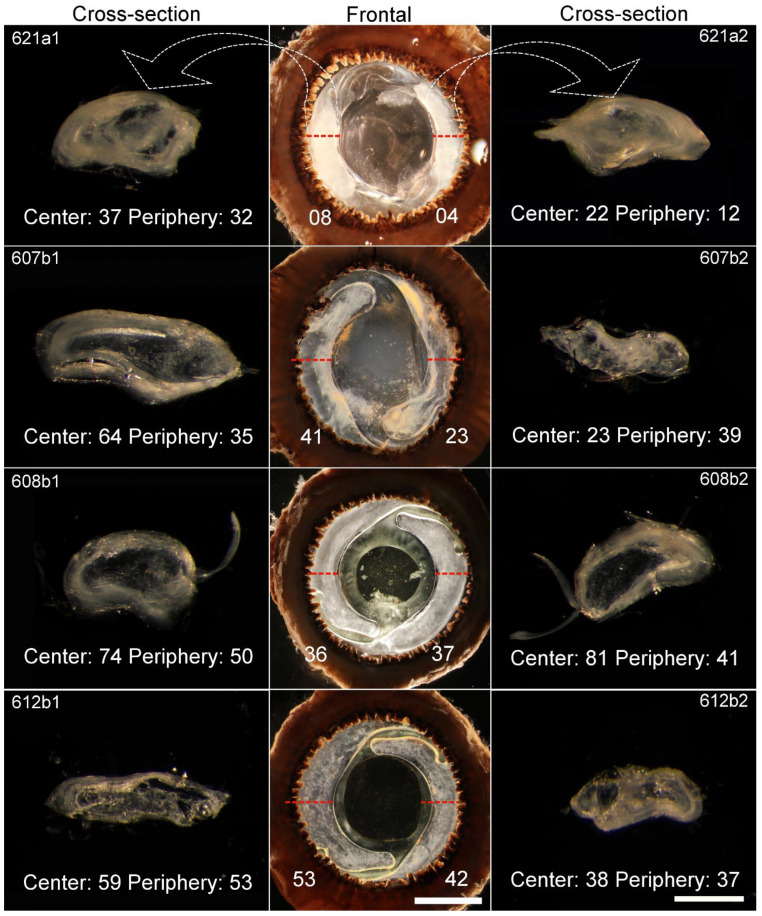
Frontal (center column) and cross-section (left and right columns) images of samples visualized with dark field illumination. The red and white dashed lines indicates where the cross-sections where made. The numerical values indicate the degree of transparency in each sample (100 = Black/transparent, 0 = White/opaque). In frontal samples it indicates the values of each side of the Soemmerring’s ring. In cross-section samples it indicates the central and peripheral values. Scale bars = 4 mm (frontal) and 800 µm (cross sections).

When viewed frontally with dark field illumination, all Soemmerring’s ring samples were apparently opaque, with low degrees of transparency, ranging from 0 to 55 (Figure 1 center column and Table 1).

However, after sectioning, samples were more transparent, with about half of the cross-section samples having areas with transparency values above 50 (Figure 1 lateral columns). The degree of transparency ranged from 0–60 at the periphery and 14–85 at the center. Furthermore, 20 of the 30 samples were more transparent in the center than at the periphery.

### 2.2. Histology

Histologically we observed different morphological factors with our immunostains and categorized them in to four levels, explained in Table 2.

**Figure 2:**
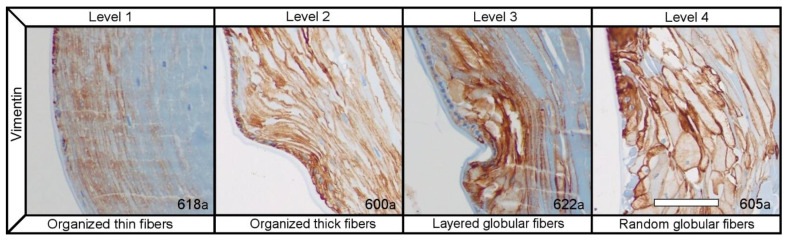
Histological sections of samples immunostained with vimentin, organized based on the level of fiber organization, based on the 4 levels explained in Table 2. Scale bar = 100 µm.

H&E, Vimentin and WGA all together allowed us to visualize the morphology and the degrees of organization of the lens fibers and epithelia throughout the samples. We differentiated 4 levels of fiber organization: level 1 (*n =* 6), which were samples with organized thin fibers; level 2 (*n =* 7), organized thick fibers; level 3 (*n =* 6), layered globular fibers; level 4 (*n =* 6), random globular fibers (Figure 2).

We also differentiated 4 levels of epithelial cell monolayer organization: level 1 (*n =* 9), which were samples with a continuous monolayer; level 2 (*n =* 2), a monolayer with additional cells; level 3 (*n =* 3), samples with more than one layer of epithelial cells and level 4 (*n =* 2), Disperse epithelial cells.

Vimentin which is expressed in lens epithelial cells and young fiber cells, also allowed us to distinguish between younger and older lens fibers. The depth of vimentin expression among the samples, ranged from 0.02–0.37 mm, with an average depth: 0.18 mm. However, there was no statistical relation between Vimentin depth and the size of samples (*p* = 0.120). But there was a statistically significant relation (*p* = 0.034) between a shallower Vimentin expression (<0.18 mm) and increased central transparency.

α-SMA which immunostains fibrotic cells, was negative in all samples (data not shown).

**Figure 3:**
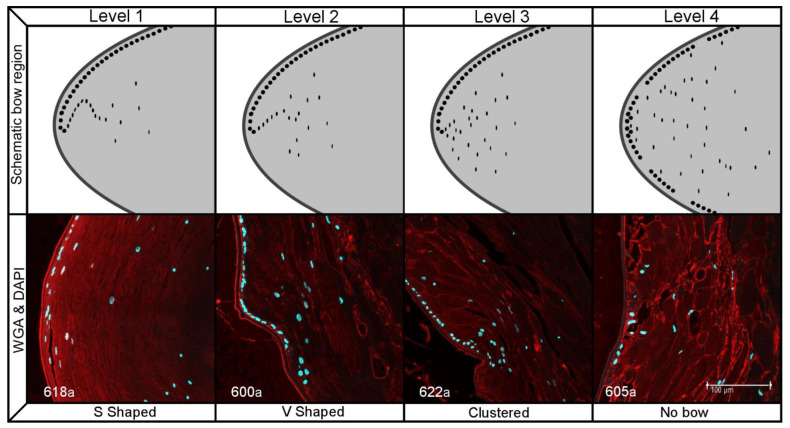
Schematic and histological representation of the 4 levels of nuclei organization at the bow. Scale bar = 100 µm.

DAPI helped us observe the distribution of the cell nuclei and categorize their level of organization at the bow region and their presence in the center of the samples. We differentiated 4 levels of nuclei organization at the bow region based on distribution (Figure 3): level 1 (*n =* 8), samples where nuclei distributed in an S shape; level 2 (*n =* 5), V shaped distribution; level 3 (*n =* 1), nuclei were clustered at the bow; level 4 (*n =* 2), no bow region was apparent. As far as the presence of nuclei in the center of the samples, 2 didn’t present nuclei in the center, 5 had a few small nuclei and 9 presented small nuclei dispersed throughout. As was to be expected, there was a significant correlation between the levels of organization of nuclei at the bow and fibers (X^2^-test *p* = 0.011).

## 3. Discussion

### 3.1. Transparency

With the results off our dark field illumination images (Figure 1 and Table 1), we could confirm that there are transparent areas within the Soemmerring’s ring, and that transparent areas are more commonly found in the center. Interestingly, the finding that the periphery is usually opaquer explains why Soemmerring’s rings are mostly seen as opaque when viewed frontally.

It is important to note that, samples might be more transparent than what we have analyzed. Previous studies have shown that lenses stored in formalin lose nearly 30% of their transparency [13]. However, since all our samples were processed the same, relative transparency between samples is maintained and this is what we compared. Furthermore, we performed a *t*-test to analyze both the postmortem time and storage time of our samples to test whether they had a significant effect on the central transparency of our samples, but neither did, with *p* = 0.264 and *p* = 0.892, respectively.

**Figure 4:**
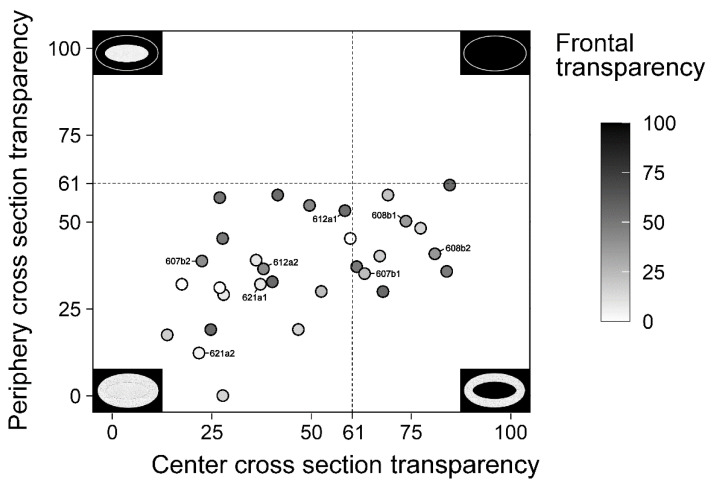
Scatter plot comparing the transparency of sample cross sections at the periphery and center. The inner color of each circle expresses the frontal transparency. The closer a value is to 0, the opaquer it is and the closer a value is to 100 the more transparent it is. 4 schematic drawings representing maximum transparency/opacity at the periphery or center can be seen at the 4 edges of the plot. For examples, the bottom left represents a sample that has a transparency of 0 both at the periphery and center. The bottom right represents a sample that has a transparency of 0 at the periphery and 100 in the center. The dashed line at 61 is the cut-off value for what we defined as transparent.

In order to better visualize these results, and analyze if there is any relation between the degrees of transparency among the different areas, we plotted the frontal, peripheral cross section and central cross section transparency values of each sample in Figure 4.

Here we can see that transparency varies most at the center, thus, in order to better analyze which factors play a role in the central transparency of our samples, we separated them in to two groups. Those with central transparency values ≥ 61 (*n =* 10) and those with central transparency values < 61 (*n =* 20) (Figure 4). The cut-off value of 61 was chosen because it is higher than any of the values seen at the periphery.

Among these two groups, we first analyzed the statistical significance of the degree of transparency of the other areas and the size of the cross-sections (Table 3).

Based on the statistics we can see that frontal opacity does not significantly relate to central transparency (*p* = 0.241), nor does the size of the growth (*p* = 0.762). Furthermore, even peripheral transparency seems not to be determined by central transparency (*p* = 0.093). This indicates that central transparency is independent of peripheral opacification.

### 3.2. Mechanisms of Transparency

**Figure 5:**
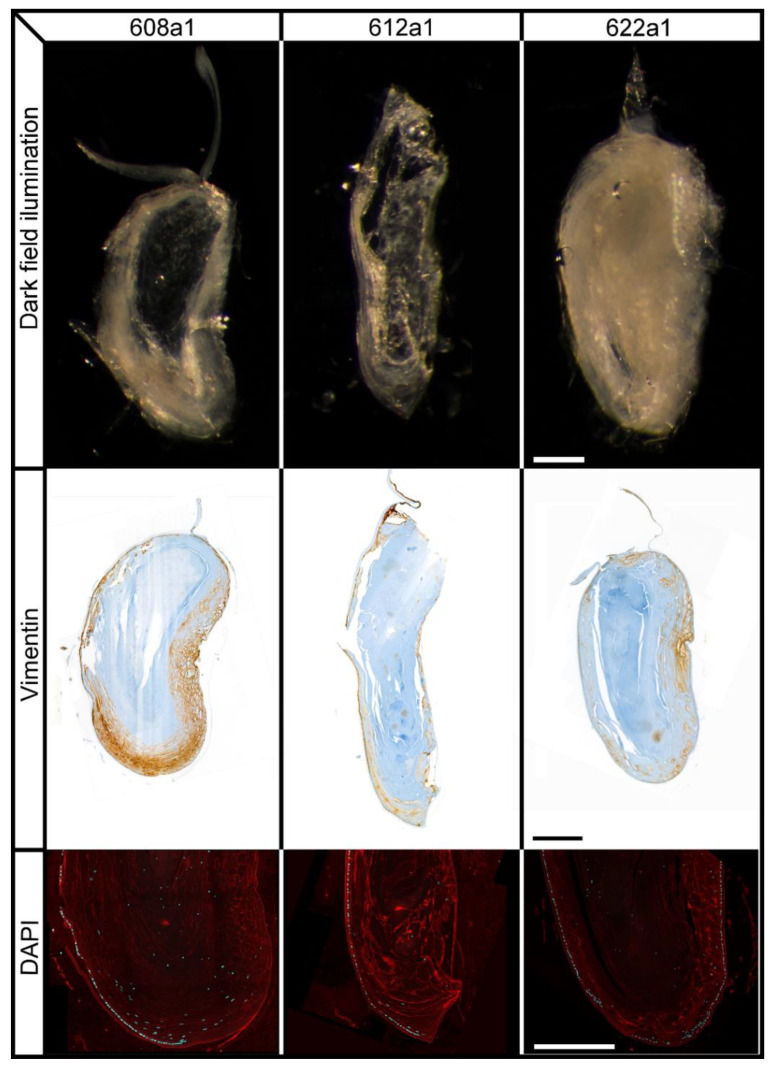
Images of 3 samples representative of our results. Cross-section dark field illumination images show different transparency/opacity distributions. Vimentin immunostaining highlights areas of developing fiber cells. DAPI highlights the distribution and size of cell nuclei in the epithelium at the lens bow and towards the center. Scale bars = 300 µm.

However, how some areas become transparent, and why most commonly in the center is not so readily apparent (Figure 5). According to the literature, transparency in the normal lens is obtained and maintained by various means. For one, lens fiber cells organize in layered concentric rings, reducing light scattering caused by the differences in refractive index between fiber membranes and cytoplasm [4,5,14,15,16]. Furthermore, lens fiber cells are tightly packed together, reducing intercellular spaces, further reducing light scatter [4,17,18]. Another mechanism is that as lens fiber cells mature, they lose their organelles and nuclei, thus eliminating light scattering elements towards the center of the lens [19,20,21].

Any or all of these mechanisms could influence the transparency of Soemmerring’s rings. In relation to these mechanisms, we have analyzed four morphological factors, the organization of lens fiber cells, the organization of the lens epithelial monolayer, the organization of nuclei at the bow region and the presence of nuclei at the center of the Soemmerring’s ring (Table 4).

There was a significant relation (*p* = 0.049) between the degree of lens fiber cell organization and the transparent center group, as was to be expected based on the literature [4,5,14,15,16,22]. However, there wasn’t a significant difference (*p* = 0.487) in the degree of organization of the lens epithelial cells between the groups as both tended to have relatively well-maintained lens epithelial cell monolayers. There also wasn’t a significant difference (*p* = 0.236) in the organization of cells at the bow region, since over 80% of samples presented a clearly defined bow region and most tended to be well organized. This could be due to the fact that in current cataract surgery, the equatorial lens capsule and the separation between the anterior and posterior chamber is maintained. Since, in the normal lens it has been shown that, the ocular media and a gradient of fibroblast growth factor between the anterior and posterior capsule aid lens epithelial cells in organizing and developing the bow region at the equator [3,23,24,25].

On the other hand, we noted that the distribution and presence of nuclei in the center did not vary significantly between groups (*p* = 0.688), and all samples tended to have dispersed nuclei present in the center. This could be explained by the fact that lens fiber nuclei degradation is the final part of terminal lens fiber maturation [19,20]. Furthermore, since the complete degradation of organelles, including the nuclei, is important in developing the transparent organelle free zone [21,26,27], this could be a reason why complete lens transparency is not achieved in the center of our samples.

In order to further study the effects of lens fiber cell maturation, we analyzed the vimentin immunostains of our samples, since vimentin is expressed in lens epithelial cells and young fiber cells but not mature fiber cells [28,29,30]. Thus, by analyzing the area within the sample not immunostained with vimentin, we could calculate the percentage of the sample area composed of mature fibers. Specifically, when comparing the percentage of the area of the cross-section composed of mature lens fiber cells, in transparent center samples (average 76%) vs. opaque center samples (average 62%). We observed that there was a statistically significant (*p* = 0.024) relation between a higher percentage of mature lens fiber cells and increased central transparency. Furthermore, the difference in maturity between the central and peripheral fiber cells could also explain the difference in transparency between these two areas. As new fiber cells grow around these more mature central fiber cells, these could be packed more tightly together, reducing the intracellular space and increasing central transparency. This could be corroborated by the lack of visible cell membranes in the center of histology samples when compared to the periphery [5] (Figure 5).

Furthermore, in order to analyze whether the lack of transparency in some of our samples was due to fibrotic cells, we used α-SMA, which is expressed in fibrotic cells and cells undergoing epithelial to mesenchymal transition. However, all samples were negative, this together with the results of our vimentin immunostains, suggested that all cells within our samples were either epithelial cells or lens fiber cells.

One question we could not answer was whether there was any correlation between the Soemmerring’s rings features we observed, and the primary cataract that were surgically extracted. It could be that similar underlying systemic issues resulting in the primary cataract, may also drive the type of resultant Soemmerring’s rings and hence their level of transparency. However, we could not study this, since we did not have access to the clinical opthalmological history of donors.

On a final note, our study does not attempt to address all the mechanisms that contribute to lens transparency, but our results do support the work of other researchers in the lens regenerative field [11,12,31,32,33,34,35,36,37], helping to increase the knowledge in this new field.

## 4. Materials and Methods

### 4.1. Tissue

Soemmerring’s rings were extracted from 34 human donor eye globes from the “Banc d’Ulls per a Tractaments de Ceguesa” over the course of a year. Only human eye globes classified as non-suitable for transplantation were used. Written informed consent for the removal and use of the eye globes for diagnostic and research purposes was obtained from donors and/or relatives. This experimental study follows the tenets of the Declaration of Helsinki and was approved by the Barraquer medical research ethics committee (CEIm).

**Figure 6:**
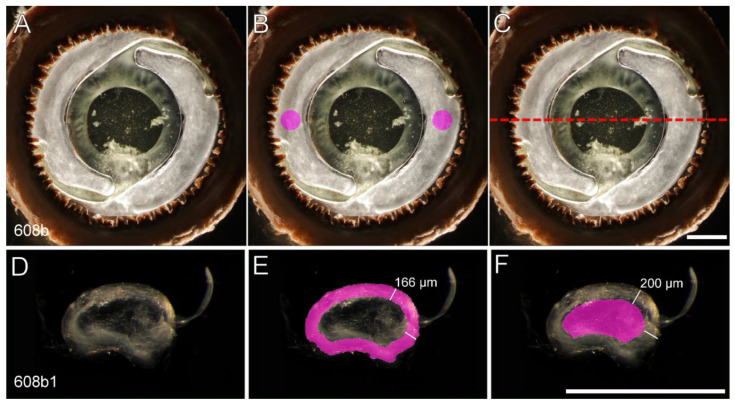
Frontal (**A**–**C**) and cross section (**D**–**F**) darkfield images of a sample (608b). (**B**) highlights (magenta) the 1 mm diameter areas analyzed for frontal transparency. (**C**) The red dashed line indicates where the cross-section was made. (**E**,**F**) highlight the two distinct areas analyzed for cross section transparency, (**E**) the periphery and (**F**) the center. Scale bars = 2 mm.

Following extraction, Soemmerring’s rings were photographed frontally (Figure 6A), with an operation microscope using dark field illumination (Supplementary Figure S1). Afterwards, samples were fixed in 4% paraformaldehyde for a week and then embedded in paraffin.

Once all samples had been collected, we discarded 10 contralateral samples randomly and 8 samples had to be discarded for either not having a growth that completely surrounded the IOL or due to a lack volumetric growth. Thus, our study was based on the 16 remaining Soemmerring’s rings (Table 5).

These samples were cut in half through the thickest part of the Soemmerring’s ring (Figure 6C). One half was kept in the paraffin block for future histological analysis and the other half was removed from paraffin, deparaffinized and thickly sectioned.

In order to obtain these thick sections, we designed a custom blade holder. Which consists of two metal plates that are screwed together, holding two blades separated by a 200 µm thick piece of plastic. Since the Soemmerring’s ring is shaped similar to a doughnut, when sectioned sagittally, it results in 2 different pieces, and we analyzed each individually, in total, we obtained 30 out of 32 cross-section pieces, having lost 2 (616a2 and 622a2) due to damage when cutting.

Once the thick sections of the samples had been obtained, samples were placed in an optic petri dish and submerged in BSS for 30 min to allow them to fully hydrate. Finally, each cross-section piece was photographed using dark field illumination (Figure 6D).

### 4.2. Quantification of Transparency

In order to objectively quantify the degree of transparency, we evaluated the luminosity values of specific areas of our sample images using the GNU Image Manipulation Program (GIMP 2.10.20, Open Source, General Public License) (www.gimp.org accessed on 28 October 2020).

In our frontal images, we analyzed two circular areas of 1 mm in diameter on opposite sides of the Soemmerring’s rings (Figure 6B). All frontal photos were obtained at ×10 magnification.

Within the cross-section images, we analyzed two different areas, the periphery and the center. The periphery was defined as the area obtained when outlining the cross section and extending the selection 166 µm towards the center of the sample (Figure 6E). The center was defined as the area located 200 µm beneath the outlined cross section towards the center of the sample (Figure 6F). These areas were based on the average depth of vimentin (180 µm) in our samples and the diameter of our smallest sample (410 µm), thus ensuring that we could use the same measurement in all of our samples. All cross-section photos were obtained at ×40 magnification and at an exposure of 1/100.

Luminosity values in GIMP are obtained as numbers between 0 and 1. A value of 0 indicates a completely black area which with dark field illumination indicates no light scatter, thus that area is transparent. A value of 1 is seen as completely white, which with dark field illumination relates to a high degree of light scatter or opacification.

After obtaining these luminosity values, we converted them into a more straight forward scale. The lowest luminosity values obtained from the backgrounds, 0.004 in cross section images and 0.012 in frontal images, were given a value of 100 to indicate complete transparency. The highest values, 0.541 in cross section images and 1 in frontal images, were given a value of 0 to indicate maximum opacity. We termed this as degrees of transparency, ranging from 100 = transparent to 0 = maximum opacity.

It is important to note that due to the variation in magnification between frontal (×10) and cross-section (×40) images, the luminosity values between both image sets could not be directly compared. This is due to the fact that the larger lens has more area to collect light, thus image brightness decreases rapidly as magnification increases [38].

### 4.3. Histology

The remaining halves of all samples, still in paraffin, were histologically sectioned at 5 µm using a microtome. They were then stained with hematoxylin and eosin (H&E) and immunostained with alpha smooth muscle actin (α-SMA) and Vimentin. Vimentin was immunolabeled using mouse monoclonal antibody V9 from Ventana, Oro Valley, AZ, USA) and α-SMA was immunolabeled using mouse monoclonal antibody 14A from Cell Marque, these immunostaining procedures were performed with the BenchMark^®^ ULTRA device (Ventana Medical Systems, Inc.) following the manufacturers protocol and counterstained with Hematoxylin.

Samples were also stained with immunofluorescent wheat germ agglutinin (WGA) and 4′,6-diamidino-2-phenylindole (DAPI). Both stains were performed together by hand, WGA (L4895, Sigma-Aldrich, St. Louis, MO, USA) was applied at 10 μg/mL for 2 h and DAPI (MBD0015, Sigma-Aldrich) was applied at 1 μg/mL for 30 min. Fluorescent images were obtained with the Leica TCS-SP5 Confocal Microscope.

## 5. Conclusions

We have completed our objective and shown that there are transparent areas within the Soemmerring’s ring of adult pseudophakic eyes. However, the reasons of the differences in transparency of the different areas of the Soemmerring’s rings, especially when compared to aphakic infants is still unclear. If we can discern the reasons behind these discrepancies, maybe we could help guide the development of Soemmerring’s rings in vivo, which could lead to the possibility of lens regeneration even in adults.

## Figures and Tables

**Table 1:** Degrees of transparency, observed frontally and in the cross-section (C-S), both at the periphery and the center and the different area sizes of each sample cross section. Samples 616a2 and 622a2 C-S omitted due to damage when cutting. The degrees of transparency range from 100 = Black/transparent to 0 = White/opaque.

Sample	Degree of Transparency			C-S Area (mm^2^)
	Frontal	C-S Center	C-S Periphery	
561a 1	49	28	45	0.89
561a 2	44	50	55	1.18
563a 1	50	27	57	1.13
563a 2	54	42	58	0.94
577b 1	55	85	60	1.02
577b 2	48	84	36	0.89
590a 1	49	61	37	1.24
590a 2	55	68	30	1.33
600a 1	55	40	33	0.71
600a 2	55	25	19	1.08
605a 1	8	37	39	1.88
605a 2	0	18	32	1.50
607b 1	23	64	35	1.27
607b 2	41	23	39	0.83
608b 1	36	74	50	1.06
608b 2	37	81	41	1.05
612b 1	53	58	53	0.93
612b 2	42	38	37	0.87
613b 1	15	28	0	1.24
613b 2	15	47	19	1.30
616a 1	15	77	48	1.01
616a 2	18	-	-	-
617a 1	11	60	45	1.72
617a 2	21	69	58	1.65
618a 1	18	67	40	1.49
618a 2	23	53	30	1.45
621a 1	8	37	32	1.06
621a 2	4	22	12	1.02
622a 1	17	14	18	1.19
622a 2	14	-	-	-
625a 1	8	28	29	1.21
625a 2	1	27	31	1.21

**Table 2:** Explanation of the levels of organization, of different morphological factors, that we used to categorize and evaluate our samples. Level 1 represents the ideal organization of cells in a normal human lens. Level 4 represents a completely unorganized cell mass. Fiber organization is illustrated in Figure 2 and nuclei organization at bow illustrated in Figure 3.

Morphological Factor	Levels of Organization			
	Level 1	Level 2	Level 3	Level 4
Fiber organization	Organized thin fibers	Organized thick fibers	Layered globular fibers	Random globular fibers
Epithelial cell organization	Continuous monolayer	Monolayer with additional cells	More than one layer	Disperse epithelial cells
Nuclei organization at bow	S shaped bow	V shaped bow	clustered bow	No bow
Nuclei presence at center	No nuclei in center	Few small nuclei	dispersed nuclei	Many large nuclei

**Table 3:** Comparison of different morphological results among our samples when separating them in to two groups based on the central transparency of their cross-sections. The mean results of transparency (ranging from 0 = most opaque sample to 100 = Transparent) and cross section area size were compared. Statistical significance of mean results was calculated with *t*-Test.

	Cross-Section Center Transparent	Cross-Section Center Opaque	*p* Value
Central cross-section transparency (0…100)	73	35	
Peripheral cross-section transparency (0…100)	44	34	0.093
Frontal transparency (0…100)	36	28	0.241
Cross-section area size (mm^2^)	1.20	1.17	0.762

**Table 4:** Comparison morphological organization among our samples when separating them in to two groups based on the central transparency of their cross-sections. The median levels of organization (Level 1…4) of the different morphological factors of each sample group were compared. Statistical significance of median results was calculated with the Kruskal-Wallis test.

	Cross-Section Center Transparent	Cross-Section Center Opaque	*p* Value
Fiber organization (Level 1…4)	1	3	0.049
Lens epithelial cell organization (Level 1…4)	1	2	0.487
Nuclei organization at bow (Level 1…4)	1	2	0.236
Nuclei presence at center (Level 1…4)	3	3	0.688

**Table 5:** Tissue donor information. Median age 91 years old, Range 54–102. Median post mortem time 96 h, Range 24–144.

Sample	Age	Sex	Post Mortem Time (h)
561A	83	F	120
563A	91	F	120
577B	92	F	144
590A	102	F	24
600A	80	M	144
605A	96	F	144
607B	79	M	96
608B	80	F	96
612B	92	F	48
613B	92	F	48
616A	91	F	96
617A	89	M	24
618A	94	F	72
621A	92	F	120
622A	54	M	120
625A	74	M	96

## Data Availability

Not applicable.

## Supplementary Materials

The following supporting information can be downloaded at: https://www.mdpi.com/article/10.3390/ijms232113294/s1.
