# Supplementary material for: Soemmerring’s Rings Developed around IOLs, in Human Donor Eyes, Can Present Internal Transparent Areas
Source: Int J Mol Sci. 2022 Oct 31;23(21):13294. doi: 10.3390/ijms232113294 (PMC9656497; doi:10.3390/ijms232113294)
Supplement: Supplementary file 1 [file ijms-23-13294-s001.zip › ijms-1940837-supplementary.pdf]

Supplementary Materials:

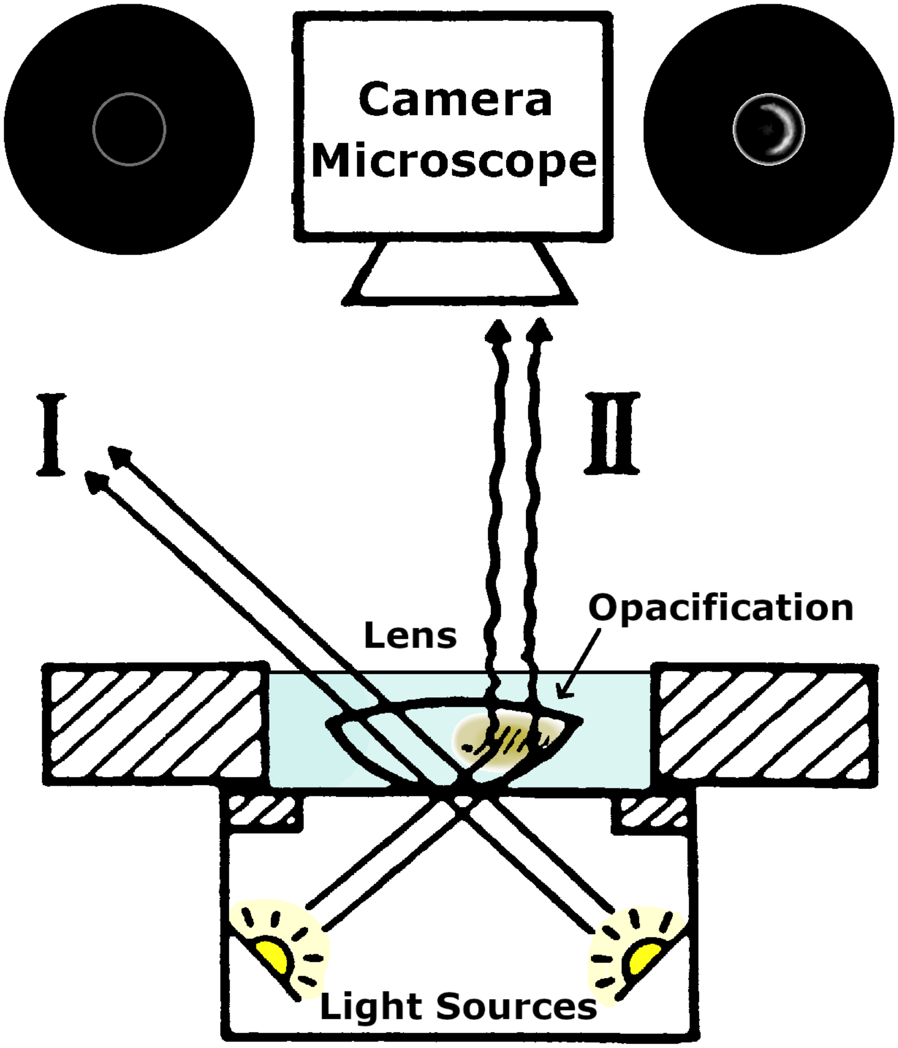

Figure S1. Schematic representation of how transparency is observed with darkfield illumination. I) Shows how when light travels through a transparent medium or sample, it is not affected nor deflected toward the microscope or camera. Thus, as seen in the top left corner, transparent lenses only show a vague outline in a darkfield. II) Shows how when light travels through an opaque medium or sample, it is partially deflected toward the microscope or camera. Thus, as seen in the top right corner, the opacifications are clearly seen. (Adapted from Söderberg et al. 1990).
